# An antennal carboxylesterase from *Drosophila melanogaster*, esterase 6, is a candidate odorant-degrading enzyme toward food odorants

**DOI:** 10.3389/fphys.2015.00315

**Published:** 2015-11-05

**Authors:** Thomas Chertemps, Faisal Younus, Claudia Steiner, Nicolas Durand, Chris W. Coppin, Gunjan Pandey, John G. Oakeshott, Martine Maïbèche

**Affiliations:** ^1^Sorbonne Universités UPMC - Univ Paris 06, Institut d'Ecologie et des Sciences de l'Environnement de Paris, INRA, CNRS, IRD, UPECParis, France; ^2^Commonwealth Scientific and Industrial Research Organisation (CSIRO) Land and Water FlagshipCanberra, ACT, Australia; ^3^Research School of Chemistry, ANU College of Physical and Mathematical Sciences, Australian National UniversityCanberra, ACT, Australia

**Keywords:** carboxylesterase, olfaction, odorant-degrading enzyme, *Drosophila melanogaster*, enzyme activity assays, electroantennogram, behavior

## Abstract

Reception of odorant molecules within insect olfactory organs involves several sequential steps, including their transport through the sensillar lymph, interaction with the respective sensory receptors, and subsequent inactivation. Odorant-degrading enzymes (ODEs) putatively play a role in signal dynamics by rapid degradation of odorants in the vicinity of the receptors, but this hypothesis is mainly supported by *in vitro* results. We have recently shown that an extracellular carboxylesterase, esterase-6 (EST-6), is involved in the physiological and behavioral dynamics of the response of *Drosophila melanogaster* to its volatile pheromone ester, *cis*-vaccenyl acetate. However, as the expression pattern of the *Est-6* gene in the antennae is not restricted to the pheromone responding sensilla, we tested here if EST-6 could play a broader function in the antennae. We found that recombinant EST-6 is able to efficiently hydrolyse several volatile esters that would be emitted by its natural food *in vitro*. Electrophysiological comparisons of mutant *Est-6* null flies and a control strain (on the same genetic background) showed that the dynamics of the antennal response to these compounds is influenced by EST-6, with the antennae of the null mutants showing prolonged activity in response to them. Antennal responses to the strongest odorant, pentyl acetate, were then studied in more detail, showing that the repolarization dynamics were modified even at low doses but without modification of the detection threshold. Behavioral choice experiments with pentyl acetate also showed differences between genotypes; attraction to this compound was observed at a lower dose among the null than control flies. As EST-6 is able to degrade various bioactive odorants emitted by food and plays a role in the response to these compounds, we hypothesize a role as an ODE for this enzyme toward food volatiles.

## Introduction

Our understanding of the molecular basis of insect olfaction has improved greatly over the last few years, in large part through the application of modern genomic technologies and advances in associated physiology. Much of the focus has been on the molecular events occurring during early olfactory processing (i.e., within the olfactory organs, also called perireceptor events Getchell et al., 1984). The steps by which odorants are bound by Odorant-Binding Proteins (OBPs) and transported to the olfactory receptors (ORs), and the ORs then activated, are now well documented (reviewed in Leal, 2013). However, the subsequent step of odorant inactivation that sustains the kinetics of the olfactory system response is still not well understood. Two sets of hypotheses are still under debate: one proposes that odorant-degrading enzymes (ODEs) are principally responsible for the rapid degradation of the odorant molecules, on a millisecond timescale (Vogt and Riddiford, 1981; Ishida and Leal, 2005; Chertemps et al., 2012), while the other invokes more complex processes also involving OBPs, ORs, or as yet unknown scavenger molecules (reviewed in Rützler and Zwiebel, 2005; Kaissling, 2009, 2014).

*In vitro* experiments have demonstrated that several important insect pheromones can be rapidly degraded by candidate ODEs belonging to various detoxification enzyme families, including esterases, cytochromes P450s, aldehyde oxidases, and glutathione S-transferases (reviewed in Vogt, 2005; Leal, 2013). Given the diversity of detoxification enzymes expressed in insect antennae (reviewed in Siaussat et al., 2014) and the variety of their potential physiological roles, functional characterizations of particular candidate ODEs are still relatively scarce. However, several antennal esterases from a range of species have now been shown *in vitro* to efficiently degrade particular sex pheromones (Durand et al., 2011; Leal, 2013; He et al., 2014a,b) and plant volatiles (Durand et al., 2010; He et al., 2014a,b). In *Drosophila melanogaster*, esterase 6 (EST-6) has been reported to degrade the pheromone cis-vaccenyl acetate (CVA; Mane et al., 1983) and more recently, a protein encoded by a duplication of the *Juvenile hormone esterase* gene (*Jhe-dup*) has been shown to hydrolyse various ester odorants for this species (Younus et al., 2014).

We have previously used comparisons between *D. melanogaster* strains carrying *Est-6* wild-type vs. null alleles (on the same genetic background) to show that EST-6 plays a role in the physiological and behavioral responses of *D. melanogaster* males to CVA (Chertemps et al., 2012). This supports the *in vitro* evidence above that this enzyme is an ODE in male antennae. Also consistent with this evidence, transcriptomic analysis of the olfactory organ shows *Est-6* and *Jhe-dup* are the most highly expressed of all the esterase genes in the antennae of this species (Younus et al., 2014).

Intriguingly however, we also find that EST-6 is widely distributed within the third antennal segment, including in sensilla tuned to other odorants, in addition to those sensitive to CVA (Chertemps et al., 2012). This suggests that EST-6 could play a broader role in the antennae than CVA processing, perhaps functioning as an ODE for other bioactive ester volatiles. *D. melanogaster* is indeed known to detect a large number of volatile esters, although their possible functions and ecological relevance are still under investigation (Mansourian and Stensmyr, 2015).

To explore this issue further, we have produced recombinant EST-6 protein using the baculovirus system and characterized its activity against eight volatile esters produced by decomposing fruits and other plant tissues (Stensmyr et al., 2003). We find that it can efficiently process most of them *in vitro*. The physiological responses of the antennae to these compounds were therefore then measured by electroantennography (EAG) on *Est-6* wild-type vs. null flies (on the same genetic background, as above). Consistent differences between the two strains were found for the six compounds for which the recombinant EST-6 had the greatest activity. For one of these, pentyl acetate, which was the strongest odorant, dose-response studies revealed that the repolarization dynamics were also modified at low doses. Behavioral studies using pentyl acetate as the bioactive molecule confirmed that EST-6 is indeed involved in the perception of this compound. These data suggest that EST-6 may function as an ODE for a variety of bioactive volatile esters in this species.

## Materials and methods

### Fly strains

The three strains used in this study were described in full in Chertemps et al. (2012). One is an *Est-6* null mutant strain (*Est-6*°; Bloomington stock 4211), where EST-6 expression is abolished, and the other is a rescue strain, *Est-6*^+^, which has the same genetic background as the *Est-6*° strain but with a fully functional *Est-6* copy inserted independently. *Canton-S* (*CS*) flies were also used as a second wild-type strain in the study of pentyl acetate responses.

All flies were raised at 25°C on standard yeast/cornmeal/agar medium in a 12-h light/12-h dark cycle, with 50–60% relative humidity.

### Assays of EST-6 activity

Three acetate esters, two propionate esters, one butyrate ester, and two methyl esters of mid-long chain fatty acids (Table 1) were tested. Octyl acetate, methyl decanoate, and methyl myristate are odorants produced by green plant tissue and the others are volatile products of rotting fruit. All eight esters were purchased in the highest available purity from Sigma Aldrich or, in the case of heptyl propionate and octyl propionate, Vigon International (USA).

**Table 1:** ***In vitro* activities of recombinant EST-6 toward the eight esters tested**.

	**Compound**	**Specificity activity**	**Kinetics**			**OR**
		**(S^−1^)**	**Kcat** **(S^−1^ ± SE)**	**Km** **(Mm ± SE)**	**Specific constant** **(M^−1^s^−1^)**	**(sensillar type)**
Good substrates	Octyl propionate	268.6	4519 ± 1683	3.16 ± 1.05	1.43 × 10^6^	Unknown
	Hexyl propionate	210.9	≥21,304	≥20	1.07 × 10^6^	Unknown
	Heptyl acetate	115.9	≥11,704	≥20	5.85 × 10^5^	67b, 13a, 92a (basiconic)
	Octyl acetate	83.3	≥8412	≥20	4.21 × 10^5^	45a, 35a (coeloconic)
	Pentyl acetate	61.9	969 ± 215	2.93 ± 0.49	3.30 × 10^5^	47a, 35a, 85c, 85b, 98a,22a, 67a (ab5B ab3B)
Poor Substrates	Propyl butyrate	18	1671 ± 748	18.33 ± 8.10	9.12 × 10^4^	19a (at3)
	Methyl decanoate	4.4	≥447	≥20	2.24 × 10^4^	Unknown
	Methyl myristate	3.1	≥309	≥20	1.54 × 10^4^	88a (at4c)

*The OR and sensillar types were taken from the OR response data bases (http://neuro.uni-konstanz.de/DoOR/default.html and http://neuro.uni-konstanz.de/DoOR/2.0/) and from Dweck et al. (2015)*.

A wild-type form of EST-6 (EST-6^F^; from strain Sengwa 24; accession KR014246) was expressed commercially (Genscript, USA) behind its own signal peptide using the BacuVance™ baculovirus expression system. An inactive EST-6 was expressed in the same way as a negative control; the gene for this was identical to that above except that the catalytic Ser^209^TCC codon was changed to Gly^209^GGG. Both proteins were concentrated ~10-fold, by passing the media through a 30 K Amicon filter. The titer of the active variant was determined using the fluorometric methods of Coppin et al. (2012).

The activity of EST-6 toward the test odorants was monitored in triplicate by gas chromatography/mass spectrometry (GC-MS) assays of substrate loss using methods modified from those described in Jackson et al. (2013). Each reaction mixture (200 μl) consisted of odorant substrate (200 μM), enzyme (0.2–3.6 nM), BSA (5 μg, for enzyme stability), and ethanol (5% v/v) in 25 mM Tris-HCl buffer (pH 8.0). Reactions were stopped by the addition of 0.5 volumes of hexane at intervals from 3 min to 20 min. The upper hexane layer was removed from the vial and analyzed by GC-MS (7890 Series, Agilent Technologies, USA) on a J&W DB-WAX column (30 m × 0.25 mm × 0.25 μm, Agilent Technologies, USA) with He (2 ml/min) as the carrier gas. The oven temperature was initially set at 50°C for 2 min and then subsequently increased over a gradient of 10°–275°C and held for 10 min. The injector and detector temperature was set at 250°C with a 10:1 split ratio.

*K*_m_-values were determined using the competitive inhibition method with 4-nitrophenyl acetate as substrate as described in Younus et al. (2014). This assay circumvents technical challenges that make the direct determination of the Michaelis–Menten kinetics impossible in the GC-MS assay above. It does this by treating the odorant ester as a competitive inhibitor of a chromogenic ester substrate for which a more facile continuous microplate assay exists. The inhibition constant, *K*_*i*_, of the “inhibitor” is equivalent to its Michaelis constant, *K*_*m*_ (Cornish-Bowden, 1995; Eisenthal et al., 2007). With the data on *K*_*m*_ and the activity at a single known substrate concentration (from the GC-MS method), the Michaelis–Menten equation can then be used to derive *K*_*cat*_.

### EAG of antennal responses to various esters

EAG was carried out to compare the responses of *Est-6*° and *Est-6*^+^ males to the eight esters above. Recordings were performed on a pClamp 10 (Molecular Devices) at 22°C on 5-day old males previously kept in individual tubes, as described in Chertemps et al. (2012). Antennae were first stimulated for 3 s with each ester (all with >95% purity, diluted 1:1000 in paraffin oil, excepting methyl myristate which was diluted in hexane). Pure hexane (>98% purity, Carlo-Erba) and paraffin oil (Sigma Aldrich) were used as negative controls. Propionic acid (Sigma Aldrich), which is not hydrolysed by esterases and is detected by another type of sensilla (Rytz et al., 2013), was used as positive control (ddH_2_0 as solvent). To further analyse the antennal responses to pentyl acetate, shorter stimulations (0.5 s) at the same dose of odorant were also performed following the same protocol. In addition, the responses to various doses (10^−4^ up to 10^−2^) of this compound were also recorded. Stimulus cartridges were changed between the tests on different insects.

Several parameters were measured. The peak amplitudes of EAG responses were measured at the maximum negative voltage deflection from the baseline (max amplitude, in millivolts) and reflect the intensity of the responses. It is generally accepted that such EAG amplitudes represent the sum of the generator potentials created by individual receptors' neurons within all the responsive sensilla carried by the antennae (Haase et al., 2011; Kaissling, 2014). The dynamics of the repolarization during and after the stimulation was estimated by three parameters: (i) a repolarization rate during the stimulation, which was calculated as [(maximum amplitude of depolarization – amplitude of depolarization at the end of stimulation)/maximum amplitude] × 100; (ii) the time at which 3/4 of maximum amplitude was recorded in seconds (3/4 repolarization time), and (iii) the value of the EAG decay slope (mV/s).

### Behavioral responses to pentyl acetate

Flies were maintained on standard yeast/cornmeal/agar medium at 25°C in a 12-h light/12-h dark cycle, 50–60% relative humidity. Newly enclosed male flies were collected and aged for 6–8 d, then wet-starved for 5 h before testing. Choice tests were then performed to assess the responses of the flies to pentyl acetate in a two-choice T-maze apparatus adapted from Stensmyr et al. (2003). All tests were performed at 25°C with 50–60% humidity and under dim red light to exclude visual effects. Responses to the control odorant propionic acid, known to trigger attraction (Knaden et al., 2012), were measured in the same conditions.

The T-maze was made of three 1 mL, 6 cm long pipettes connected with a Three-way splitter (E765.1, Roth) and tightly sealed with parafilm. Solutions of pentyl acetate ranging from 10^−1^ (i.e., 920 ng of pentyl acetate) up to 10^−9^ were prepared. For propionic acid, solutions ranging from 10^−2^ (i.e., 99 ng of propionic acid) up to 10^−8^ were used. Ten microliters of the corresponding solution was applied to a piece of filter paper (5 × 5 mm), which was then placed in a 1.5 mL tube (no. EA83.1; Roth). This tube was then joined to one arm of the T-maze, and a control tube with the solvent (i.e., paraffin oil) on an equivalent piece of filter paper was then embedded on another arm. A single male was introduced into the third arm of the T-maze by gentle aspiration and allowed to move through the maze for 2 min, after which the arm in which it was located was recorded. At least 70 replications with different males for each strain and pentyl acetate or propionic acid concentration were performed and the position of the stimulation and control arm was alternated between trials. The response index (RI) was calculated as (number of flies in odorant arm/total number of tested flies). An RI of 1 represents full attraction, a value of 0 represents full avoidance, and 0.5 indifference to the odor.

### Statistical analysis

All statistical analyses were performed using the Graphpad® Prism 5 software. For the EAG study with the eight esters, Mann–Whitney or Student's *t*-tests were used for pairwise comparisons depending on the distribution of the EAG variables. For the EAG study with pentyl acetate, ANOVA analyses (both parametric and non-parametric according to the data distribution) followed by *post-hoc* tests (Tukey or Dunns), were performed. For behavioral analysis, the Wilcoxon signed rank test was used to test data from the behavioral choice experiment against the null hypothesis of indifference to the odorant and non-parametric ANOVA followed by Dunns *post-hoc* tests were used for comparisons between genotypes.

## Results

### *In vitro* EST-6 activity toward various esters

Recombinantly expressed EST-6 showed detectable specific activity against all eight naturally occurring esters tested. However a strong preference for acyl groups containing no more than three carbons was evident; propyl butyrate yielded activities at least three fold lower than the five acetate and propionate substrates, while the values for methyl decanoate and methyl myristate were several fold lower again (Table 1). Consistent with expectations for an enzyme with a relatively broad substrate range, the kinetic data showed high *K*_*m*_-values (in the mM range) for all substrates, indicating relatively loose enzyme-substrate affinities. However, *k*_*cat*_-values were also quite high, particularly for the substrates with the shorter acyl groups, although even methyl decanoate and methyl myristate yielded non-negligible estimates. Despite the high *K*_*m*_-values, the high *k*_*cat*_-values, and the high specificity constants (*k*_*cat*_/*K*_*m*_) that follow, would suggest that the enzyme could effectively turn over the locally high concentrations of several of the substrates that might be expected in the vicinity of the ORs in the sensilla.

### Global comparison of EAG responses to the eight esters

**Figure 1:**
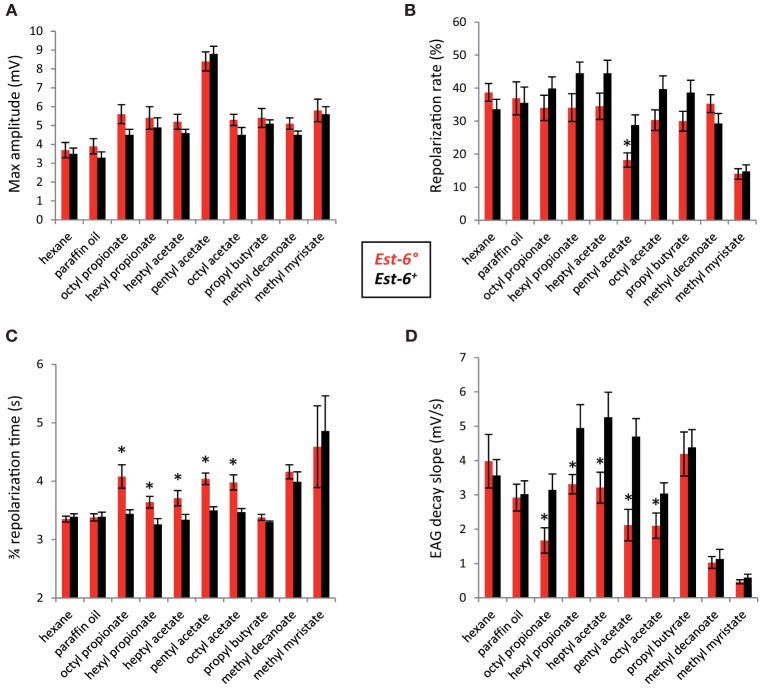
**Comparison of antennal responses to the eight esters between the two *Est-6* strains**. **(A)** Peak amplitudes; **(B)** Repolarization rates; **(C)** 3/4 repolarization times; **(D)** EAG decay slopes (mV/s). These parameters were detailed in the Materials and Methods. (Means ± sem; ^*^p < 0.05; *n* ≥ 10).

As a first step in testing whether the *in vitro* activities above were indeed relevant *in vivo*, we compared the olfactory responses of *Est-6*° and *Est-6*^+^ males to the eight esters by EAG. We used a dose of each odorant that should induce a high response and gave a relatively long stimulation (3 s) in order to represent an overstimulation of the antennae. Five of the chosen esters (pentyl, octyl, and heptyl acetate, propyl butyrate and methyl myristate) were previously known to be detected by the fly (Cobb and Dannet, 1994; Stensmyr et al., 2003; Schlief and Wilson, 2007; Dweck et al., 2015; http://neuro.uni-konstanz.de/DoOR/2.0/), with no data on the question available for the other three (Table 1). Moreover, the responses to octyl and heptyl acetate have only been reported for larvae and at the behavioral level (Cobb and Dannet, 1994). Methyl myristate has been shown recently to be involved in short-range attraction in both sexes (Dweck et al., 2015). Here, we first showed that all eight of the compounds tested were detected by the antennae of the different strains (Figure 1A) and could thus be possible odorants. The maximum EAG amplitude values for both strains were indeed statistically different from the responses induced by the paraffin oil and hexane controls, the latter two being identical between the two strains (*p* < 0.05, Student's *t*-test). Pentyl acetate induced the strongest EAG responses (max amplitude >8 mV) in both strains.

No variation in the peak amplitude of depolarization was observed between the two strains for any of the compounds tested (Figure 1A), suggesting that EST-6 does not affect the intensity of the response. However, the repolarization dynamics during the stimulation are significantly different in the case of pentyl acetate (repolarization rate of 18.2 cf 28.8% in *Est-6*° and *Est-6*^+^ males, respectively; Figure 1B). This parameter was also lower in *Est-6*° males for the other esters for which EST6 had good activity (referred as “good substrates” in Table 1) but with no statistically significant differences. After stimulation, the 3/4 repolarization time and the decay slope values also differed significantly between the two strains for all these five compounds (Figures 1C,D), whereas the latter measures of the dynamics of the response were not affected for the three other compounds. Responses of the different strains to the control odorant propionic acid were similar (Figure S1).

### Further analysis of EAG responses to pentyl acetate

**Figure 2:**
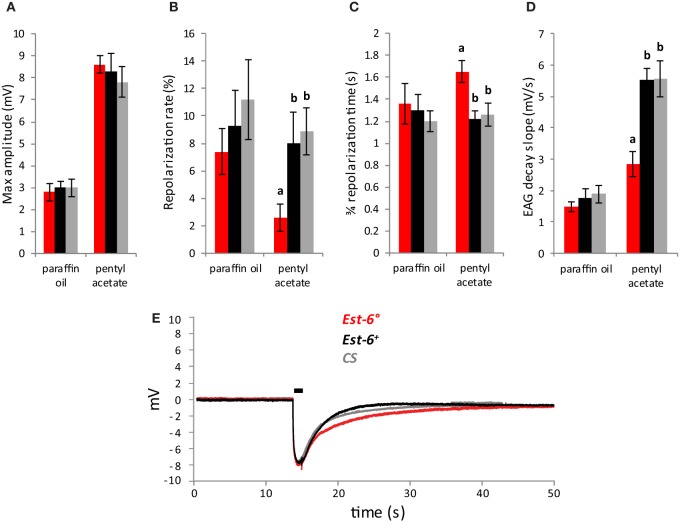
**Antennal responses to pentyl acetate after a 0.5 s stimulation (10^−3^ dilution)**. **(A)** Peak amplitudes; **(B)** Repolarization rates; **(C)** 3/4 repolarization times; **(D)** EAG decay slopes (mV/s) **(E)**; Average EAG plots from null mutant (*Est-6*°*)*, rescue (*Est-6*^+^*)*, and *CS* flies. Horizontal bar indicates the duration of stimulus delivery. Data notated with different letters are significantly different (Means ± sem; *p* < 0.05; *n* ≥ 10).

**Figure 3:**
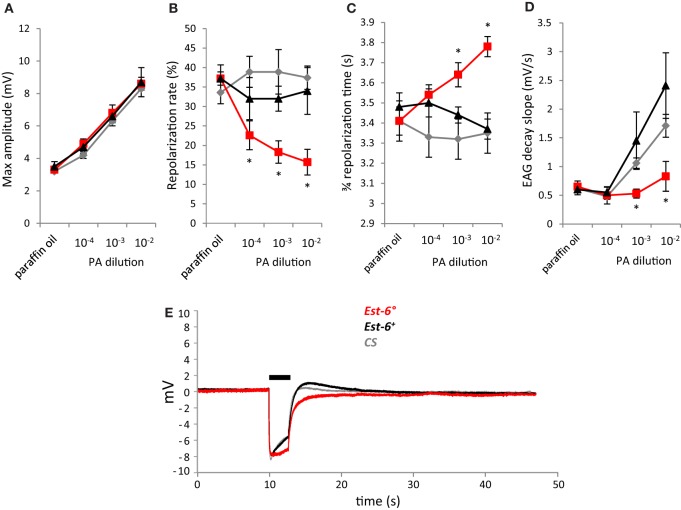
**Antennal responses to serial dilutions of pentyl acetate (PA) after a 3 s stimulation**. **(A)** Peak amplitudes; **(B)** Repolarization rates; **(C)** 3/4 repolarization times; **(D)** EAG decay slopes (mV/s); **(E)** Average EAG plots from null mutant (*Est-6*°*)*, rescue (*Est-6*^+^*)*, and *CS* flies after a stimulation dose of 10^−3^. Horizontal bar indicates the duration of stimulus delivery. (Means ± sem; ^*^p < 0.05; *n* ≥ 6).

As pentyl acetate elicited the strongest antennal responses (Figure 1A), recordings with this compound were also performed using short stimulation duration and different doses. A second wild-type strain, *CS*, was also used in these experiments. The differences in the responses of the null and wild-type strains were again seen when the antennae were stimulated with pentyl acetate at 10^−3^ for a shorter period (0.5 cf. 3.0 s, Figure 2). Compared to the *Est-6*^+^ and *CS* males, the *Est-6*° males showed a slower repolarization rate during the stimulation (2.6 vs. 8%, and 8.9%, respectively), and a slower repolarization, further supporting a role for EST-6 in the temporal dynamics of antennal responses to this compound. Responses to several doses of pentyl acetate were also compared following 3 s stimulations (Figure 3). The peak amplitude was again not modified but the repolarization dynamics were altered even at the lowest dose (10^−4^ dilution).

### Behavioral responses to pentyl acetate

**Figure 4:**
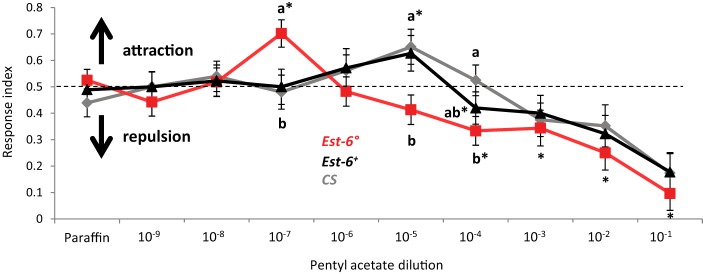
**Behavioral responses to pentyl acetate**. Fly behavior was tested in a T-maze setup and the odorant was presented over a wide range of concentrations. The behavioral data are based on the response of a total of 2100 tested flies with at least 70 flies per genotype/condition. A response index (RI) of 1 indicates attraction, 0 represents avoidance, and 0.5 indifference to the odor. Asterisks indicate *RI*-values significantly different from 0.5 (Wilcoxon rank test, *p* < 0.05). Data notated with different letters are significantly different (ANOVA followed by Dunns *post-hoc* test, *p* < 0.05).

As pentyl acetate was the only compound tested here already known to induce a clear behavioral response in adults and as its antennal detection was clearly impaired in the *Est-6*° mutants, we performed a choice assay in order to test if the lack of EST-6 could also alter their behavioral responses. As shown in Figure 4, the overall responses of all strains to the odorant followed the pattern expected for a bioactive volatile (Stensmyr et al., 2003), with indifference to low concentrations, attraction to intermediate concentrations and repulsion to high concentrations. The attractive response was clearly modified in the *Est-6*° mutant flies, with a threshold attractive dose of 10^−7^ compared to 10^−5^ in the two control strains. No significant difference was seen between *Est-6*° and *Est-6*^+^ strains in the threshold for the repulsion response, which was at 10^−4^ dilution in both cases, even if at 10^−5^ the *Est-6*° mutant flies were already in the repulsive part of the response. The *CS* flies were repulsed at higher dose (10^−3^), a difference that could be explained by their different genetic background compared to *Est-6*° and *Est-6*^+^ flies (because of which the *Est-6*^+^ strain is a more reliable control strain.) Thus the *Est-6*° males had a 100-fold lower threshold for their attractive response to this odorant than did the *Est-6*^+^ and *CS* males and a tenfold lower threshold for their avoidance response than did the *CS* flies. Responses of the different strains to the control odorant propionic acid were similar (Figure S2).

## Discussion

High temporal resolution of the chemical signal within the olfactory system is required to allow an accurate spatial location of odorant sources by the insect. Inactivation of odorant molecules which are a few milliseconds old in the vicinity of the ORs is a necessity for the dynamics of the response. The data accumulating in the literature suggest that ODEs could be involved at least in part in odorant inactivation (reviewed in Leal, 2013). Pheromone or plant odorant degradation *in vitro* by antennal extracts or recombinant enzymes (reviewed in Jacquin-Joly and Maïbèche-Coisne, 2009; Leal, 2013), as well as *in vivo* inhibition (Chertemps et al., 2012) support this hypothesis.

Most of the data that support the role of ODEs have been obtained on extracellular antennal esterases from moths (reviewed Leal, 2013; He et al., 2014a,b) and *Drosophila melanogaster* (Chertemps et al., 2012; Younus et al., 2014). Several sets of physiological and behavioral data for this latter species have previously suggested that an antennal extracellular carboxylesterase, EST-6, acts as an ODE in the detection and perception of the volatile pheromone CVA. However, its widespread expression through the third antennal segment (Chertemps et al., 2012) suggests a more general role in odorant processing. We show here that this carboxylesterase is indeed able to degrade a range of volatile esters emitted from natural *D. melanogaster* food sources *in vitro* and that it plays a role in antennal responses to those esters. Notably, those esters which were good substrates for the enzyme *in vitro* were also those which affected antennal responses, while those which were poor substrates, such as the pheromonal compound methyl myristate (Dweck et al., 2015), did not affect antennal responses. In the case of pentyl acetate, we further show that the effects of EST-6 translate to behavioral changes in the presence of the odorant. A previous study of purified EST-6 had also shown *in vitro* activity against several other volatile esters which could be emitted by rotting fruit (e.g., ethyl acetate and butyl acetate; Danford and Beardmore, 1979). It appears that EST-6 may act as a general rather than specific ODE.

The kinetics of EST-6 toward the five preferred substrates among the eight esters studied here are in the range reported for other insect esterases that have been proposed as ODEs against their respective odorants (reviewed in Younus et al., 2014). Given the abundance of EST-6 within the antennae (Anholt and Williams, 2010), this suggests that EST-6 should be an efficient ODE for processing these odorants. Interestingly, JHEdup, the other *D. melanogaster* esterase proposed to have an ODE function, was also found to have kinetics in this range for the five acetate esters of various primary, secondary and unsaturated esters that were tested (Younus et al., 2014). And, in the case of the one substrate also tested here, pentyl acetate, its kinetics were comparable to those of EST-6. While more work is needed to establish the extent of the overlap in substrate range, the pentyl acetate results suggest that more than one esterase ODE could act on the same volatile, depending on their expression patterns within the antennae. Such an overlap could reduce the size of the *in vivo* effects evident from mutation or inhibition of any one of the ODEs in question.

Few data have been available to date on the effect of mutation or inhibition of candidate ODEs on the electrophysiological responses of insect antennae. Several pharmacological carboxylesterase inhibitors (trifluoroketones) have been used in experiments with different lepidopteran species to test whether they affected responses to pheromones, but the effects reported remain controversial (reviewed in Vogt, 2005); the targets of these inhibitors, i.e., esterases but also putatively OBPs or ORs, are still debated. In vertebrates, a potential role of esterases in the nasal mucus has also been revealed by pharmacological inhibition approaches which show that enzymatic conversion of odorants could be fast enough to affect the intensity and dynamics of the olfactory responses (Nagashima and Touhara, 2010; Thiebaud et al., 2013). More recently, we have shown that a null mutation of EST-6 in *D. melanogaster* modifies the neuronal responses of males to the pheromone CVA, prolonging the period of response, as might be expected in the absence of the relevant ODE (Chertemps et al., 2012). Similarly, our EAG data indicate that the temporal dynamics of antennal responses to various food odors that are good substrates for EST-6 are altered in EST-6 null males. A delay in signal termination was found for each of these compounds and, for pentyl acetate, a slower rate of recovery was evident even during the stimulation period (for both the stimulation periods tested).

Direct comparison between a behavioral and an electrophysiological response to an odorant should always be interpreted carefully, as the contexts of response recordings are different. These EAG results on pentyl acetate detection are nevertheless consistent with the differences we found between the two *Est-6* genotypes in the behavior induced by this odorant. We found that *Est-6*° males had lower thresholds of attractive response to this volatile than did wild-type males in our behavioral choice experiments. This mirrors our previous finding that *Est-6*° males also have lower thresholds for behavioral responses (including enhanced anti-aphrodisiac effects) to the pheromone CVA (Chertemps et al., 2012). Taken together, the data suggest that *Est-6*° flies are more sensitive to both these ester compounds. We expect that the lack of EST-6 in the mutant antennae may delay the degradation of these esters in the sensillar lymph, which in turn could delay signal termination and modulate, at least partially, the corresponding behaviors.

Deciphering the molecular mechanisms underlying the perception and the responses of the organisms is a major problem for behavioral neurosciences. If ORs are the main components of the olfactory system, a better understanding of the perireceptor events occurring within the sensillar lymph that could regulate their activities is still required. We have shown that EST-6 is able to degrade various volatile esters *in vitro* and plays a role in the response of the flies to these esters, as expected for an ODE. Further characterization of these extracellular chemosensory enzymes, both *in vitro* and *in vivo*, should provide a better understanding of how they modulate the sensory input and the dynamics of the response.

### Conflict of interest statement

The authors declare that the research was conducted in the absence of any commercial or financial relationships that could be construed as a potential conflict of interest.

## Supplementary material

Figure S1 **Antennal responses to propionic acid (10^−1^ dose) after a 3 s-stimulation. (A)** Peak amplitudes; **(B)** Repolarization rates; **(C)** 3/4 repolarization times; **(D)** EAG decay slopes (mV/s). Means ± sem; *p* < 0.05; *n* ≥ 7.

Figure S2 **Behavioral responses to propionic acid**. A response index (RI) of 1 indicates attraction, 0 represents avoidance, and 0.5 indifference to the odor. Asterisks indicate *RI*-values significantly different from 0.5. At least 70 flies per genotype/condition were tested (Wilcoxon rank test, *p* < 0.05; ANOVA followed by Dunns *post-hoc* test, *p* < 0.05).
